# Person-Centered Care in Digital Health Interventions for Chronic Diseases: A Systematic Review

**DOI:** 10.3390/healthcare14081048

**Published:** 2026-04-15

**Authors:** Adrijana Svenšek, Lucija Gosak, Tamara Trajbarič, Luka Šajher, Gregor Štiglic, Mateja Lorber

**Affiliations:** 1 Faculty of Health Sciences, University of Maribor, Žitna Ulica 15, 2000 Maribor, Slovenia; lucija.gosak2@um.si (L.G.); tamara.trajbaric1@um.si (T.T.); luka.sajher@student.um.si (L.Š.); gregor.stiglic@um.si (G.Š.); mateja.lorber@um.si (M.L.); 2 Faculty of Electrical Engineering and Computer Science, University of Maribor, Koroška cesta 46, 2000 Maribor, Slovenia; 3 Usher Institute, University of Edinburgh, 5 Little France Rd, Edinburgh EH16 4UX, UK

**Keywords:** person-centered care, chronic diseases, digital transformation, sustainable development

## Abstract

Background/Objectives: Digital health interventions are increasingly used to support person-centered care (PCC) in chronic disease management, yet it remains unclear which PCC components are most consistently enabled by digital tools and how these relate to outcomes. This study synthesized evidence on digitally supported PCC for adults with chronic conditions, examining how interventions operationalize PCC and which clinical, patient-reported, and implementation outcomes are reported. Methods: A structured literature synthesis was conducted according to PRISMA guidelines across a heterogeneous evidence base, including randomized and pragmatic trials, observational studies, qualitative studies, and systematic reviews. The review protocol was pre-registered in the Open Science Framework (OSF) Registries. Results: Across 16 included studies, digital solutions most consistently supported PCC through enhanced situational awareness via self-monitoring, strengthened partnership through two-way communication and coaching, and reinforced shared documentation through co-created health plans. Benefits were reported most consistently for process and experience outcomes, such as perceived access to support, engagement, and empowerment. Evidence for sustained long-term clinical improvements, such as glycemic control, was mixed and frequently limited by short follow-up periods and variation in intervention integration. Conclusions: Digitalization can strengthen PCC when embedded within relational care models and organizational workflows that translate patient-generated data into meaningful action. Future work should utilize clearer PCC operationalization, longer follow-up, and routine reporting of equity outcomes, alongside targeted training for healthcare professionals delivering PCC in digital encounters.

## 1. Introduction

Chronic diseases represent a major part of the health burden and require long-term management, which makes it even more important to understand precisely what they actually are [1]. Multiple definitions exist, one commonly cited definition describes chronic diseases as conditions lasting three months or longer, and adds that chronic diseases cannot be prevented by vaccines or cured by any medications [2,3]. The World Health Organization (WHO) defines chronic diseases as non-communicable conditions that are long-lasting and are divided into four types: cardiovascular diseases, cancer, chronic respiratory diseases, and diabetes [4]. Definitions and the differences between them become most noticeable when they are compared internationally. These variations in definitions do not only cause confusion among patients but also make it more difficult for them to understand and manage their own illness [2]. With the rise in chronic diseases, new challenges in managing them are also emerging. One of the solutions is proper individualized patient care, which includes addressing the patient’s specific needs and encouraging independence with the goal of enabling self-management of chronic illness [5,6]. The self-management approach brings progress, such as preventing progression, reducing complications/exacerbations, improving early detection and timely management [7,8]. For individualized care and self-management to function effectively in the long term, continuity of care is essential. Continuity in nursing care is usually defined as a longitudinal relationship between the patient and their personal physician or a member of the healthcare team [9]. Continuity itself represents an approach to care that ensures cooperation between the patient and healthcare team members and guides them toward a common goal, such as high-quality and cost-effective healthcare [10,11]. In healthcare, continuity is associated with improved preventive and chronic care, a better patient experience, and more cost-effective treatment [12]. At the same time, maintaining continuity of care increasingly relies on modern tools that support ongoing monitoring and communication. With the scientific advancement of medicine and nursing, approaches to managing and monitoring individual health status are changing and gradually becoming digitalized [13,14]. Digital healthcare offers a wide range of possibilities that will most likely improve the quality of patient care [15]. The traditional paradigm of clinical history-taking, examinations, differential diagnoses, and treatment may be enhanced through tools such as technology-supported learning, mobile applications, sensors, wearable devices (smart bracelets/watches), telehealth, and others [16]. The goal of using technology to support the management of chronic diseases is to improve the quality of healthcare while also potentially redefining the boundaries of person-centered care (PCC) [17,18]. Building on these developments, it becomes clear that the purpose of continuity, self-management support, and digital tools is ultimately to achieve high-quality PCC [19]. The term is sometimes confused with personal care; however, in this context personal care refers to technology-driven customization of care to the patient (i.e., adapting digital tools or services to individual users), whereas person-centered care emphasizes the individual’s values, preferences, goals, and active role in decision-making [20,21]. In the literature, several related terms are used to describe PCC (e.g., person-centered, patient-centered, individualized, and personalized), which reflects that the concept is discussed from slightly different perspectives [22]. Despite these terminological differences, most definitions emphasize that PCC involves systematically identifying an individual’s values, preferences, and priorities and consistently using them to guide all aspects of healthcare [23,24]. It also focuses on supporting the achievement of realistic health and life goals and is grounded in a dynamic, collaborative partnership between the patient, people who matter to them, and relevant healthcare professionals [25]. This partnership enables shared decision-making to the extent the patient wishes [23]. The purpose of PCC is to improve the safety, quality, and coordination of healthcare, as well as quality of life. However, this approach does not have universally prescribed standards or pre-agreed parameters for implementation [22,23,26,27]. Precisely because standards, parameters, and other key elements for defining PCC-related interventions are often insufficiently specified, interventions may be inaccurately evaluated and presented as PCC even when they are not [28]. To assess the effectiveness and functionality of PCC, research and clinical settings commonly use questionnaires and validated measurement scales that enable a comprehensive evaluation of different dimensions of care quality from the perspectives of both patients and healthcare providers [29,30,31]. Some researchers also argue against a single fixed PCC theory and suggest that the definition and delivery of care should be adapted to the individual’s needs. Different diagnoses may lead to different PCC-relevant outcomes, since the care plan is adjusted to the underlying cause and characteristics of the chronic condition [32,33,34].

The purpose of this systematic review is to identify digital interventions for adults with chronic diseases that incorporate PCC. The aim is to guide further development and evaluation of digital solutions and to highlight the need for additional research in this area.

Building on these developments, this systematic review aims to answer the following research questions:

1. Which digital health interventions for adults with chronic diseases explicitly incorporate PCC?
2. How is PCC operationalized within these digital environments?
3. What clinical, psychosocial, and implementation outcomes are reported in relation to digitally supported PCC?

## 2. Materials and Methods

The review followed a structured narrative systematic synthesis approach, as it includes heterogeneous evidence types (RCTs, qualitative, and systematic reviews). While reported in accordance with PRISMA guidelines, the synthesis was adapted to accommodate methodological diversity, aligning with a narrative systematic synthesis framework. The review was reported in accordance with the PRISMA guideline, and a PRISMA flow diagram was used to document the study selection process and PRISMA checklist in Supplementary Materials [35]. Eligibility was defined a priori using a PIO-informed structure (population, intervention, outcomes) [36], guided by the following question: which digital health interventions that incorporate person-centered care (I) have been identified, and how are they described in terms of person-centered care implementation (O), for adults with chronic diseases (P)?

Studies were eligible for inclusion if they were published in English, involved adults (≥18 years) with a chronic disease or long-term condition, and evaluated a digital health intervention (including, but not limited to, eHealth, mHealth, telehealth/telemedicine, mobile applications, digital technologies or digital interventions, wearables, and remote monitoring). To be included, studies also had to explicitly describe the care approach as person-centred/person-centered, patient-centred/patient-centered, individualized, or personalized care, and be reported as empirical research (quantitative, qualitative, or mixed-methods) and/or systematic reviews addressing PCC within eligible digital interventions. Studies focused on children or adolescents, or on acute conditions without a chronic disease focus, were excluded, as were publications that reported only technical development or feasibility without evaluating use in an eligible population (e.g., engineering or system design papers without health or PCC evaluation). We also excluded studies in which PCC was not explicit (i.e., person-centred/person-centered or related terminology was not stated or was not clearly linked to the intervention’s design or evaluation), and non-research publication types such as editorials, commentaries, protocols, and letters where full evaluative data were unavailable. The protocol for this systematic review was registered in the Open Science Framework (OSF) Registries to enhance transparency and is publicly available at: https://osf.io/wpc98 (accessed on 2 January 2026).

### 2.1. Search Strategy

A comprehensive electronic search was conducted in PubMed, MEDLINE, CINAHL Ultimate, Web of Science, and Scopus (up to 18 November 2025). To ensure a comprehensive retrieval, the search employed controlled vocabulary terms, such as Medical Subject Headings (MeSH) in PubMed (e.g., ‘Patient-Centered Care’, ‘Chronic Disease’, ‘Telemedicine’), alongside the core search string. Additionally, reference lists of all included studies were manually screened (snowballing) to identify any further relevant publications.

The search strategy was developed based on the research question addressing how PCC is defined and implemented in digital health interventions for chronic diseases and which outcomes are reported. The core search string was:

(“person-centered care” OR “person-centred care” OR “patient-centered care” OR “individualized care” OR “personalized care”) AND (“digital health” OR “eHealth” OR “mHealth” OR telehealth OR telemedicine OR “digital technolog*” OR “digital intervention*” OR “mobile app*” OR wearable* OR “remote monitoring”) AND (“chronic disease*” OR “chronic condition*” OR “long-term condition*” OR “non-communicable disease*”).

### 2.2. Review Process

**Figure 1:**
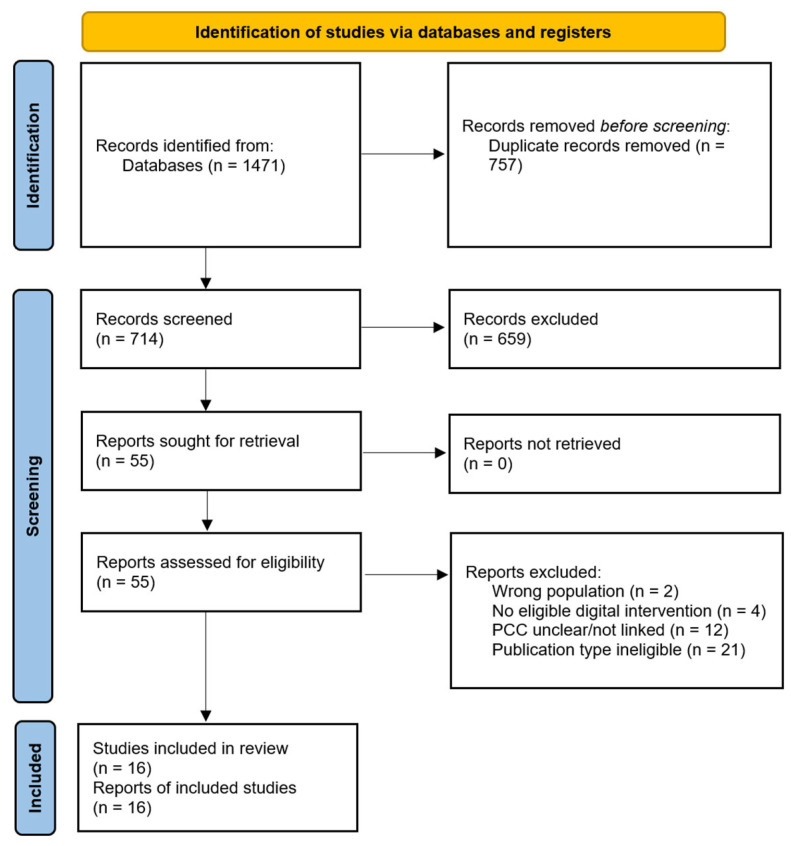
PRISMA 2020 flow diagram [36].

Using the predefined search strategy, we identified *n* = 1471 records (Figure 1). The database search yielded the following number of records: MEDLINE (*n* = 284), PubMed (*n* = 299), Web of Science (*n* = 277), Scopus (*n* = 437), and CINAHL Ultimate (*n* = 174). All identified records were exported to Rayyan [37] for screening. Deduplication was performed using Rayyan’s automated duplicate detection tool, followed by manual verification by two independent reviewers to ensure the accuracy of the final dataset. Screening was conducted independently by two reviewers using the blinding function. After removal of duplicates (*n* = 757), the remaining records (*n* = 714) were screened by title and abstract, and *n* = 659 records were excluded. Full texts of *n* = 55 articles were then assessed for eligibility. The main reasons for full-text exclusion were ineligible population (e.g., not adults with chronic disease/long-term conditions) (*n* = 2), no eligible digital health intervention (*n* = 4), PCC not explicit or not clearly linked to the intervention (*n* = 12), and ineligible publication type (e.g., protocol, commentary, editorial) (*n* = 21). Disagreements between reviewers were resolved through discussion and, when required, consultation with a third reviewer. In total, *n* = 16 studies were included in the final synthesis. Although the initial search yielded 1471 records, the rigorous application of exclusion criteria, specifically the requirement for an explicit link between PCC terminology and digital intervention, led to the final inclusion of 16 records. This small number highlights a significant gap in the literature: many digital tools support PCC principles implicitly, but few formal evaluations utilize recognized PCC frameworks.

### 2.3. Data Extraction

Two reviewers independently extracted data from all included studies and entered the information into Rayyan [37]. For each study, we extracted data in tables: bibliographic and study characteristics (author, year, country, methodology, population, digital intervention, PCC (person-centered care/person-centered principles)) and outcomes. Any discrepancies in extracted data were resolved by consensus; if consensus could not be reached, a third reviewer adjudicated.

### 2.4. Quality Assessment

Quality appraisal and data handling incorporated a qualitative component using the JBI Critical Appraisal Checklists (2019), in line with the PRISMA reporting framework [35]. This approach enabled a structured assessment of methodological quality across the included study designs and supported consistent extraction and interpretation of evidence relevant to PCC. Two reviewers independently appraised each study and subsequently compared ratings; disagreements were resolved through discussion and, where necessary, adjudication by a third reviewer. The following JBI checklists were applied according to study design: randomized controlled trials [38], non-randomized experimental studies [39], analytical cross-sectional studies [40] and cohort studies [41]. After scoring, a total score and percentage of the maximum possible score were calculated for each study. Based on [42], studies were categorized as low (60–69%), moderate (70–79%), high (80–89%), or excellent quality (>90%), and only studies meeting at least 60% of JBI criteria were eligible for inclusion in the review. In addition, the hierarchy of evidence [43] was used to contextualize the overall strength of evidence. Given the methodological heterogeneity across studies, findings were synthesized using a structured narrative approach to address the research question [44]. The methodological quality assessment informed the synthesis by ensuring that only studies meeting a minimum threshold (≥60%) were included. Findings from studies categorized as ‘High’ or ‘Excellent’ quality were given greater weight in the interpretation of clinical and psychosocial efficacy, while lower-quality studies were used primarily to illustrate emerging themes in digital PCC operationalization.

## 3. Results

### 3.1. Study Characteristics and Intervention Modalities

The synthesis included 16 sources published between 2011 and 2025, reflecting more than a decade of evolution in digital health and person-centered care. The evidence base was methodologically diverse and included systematic reviews (*n* = 3; [45,46,47]), randomized controlled trials (*n* = 2; [48,49]), qualitative studies (*n* = 5; [50,51,52,53,54]), feasibility and pilot studies (*n* = 3; [55,56,57], and observational or non-randomized quantitative studies (*n* = 3; [58,59,60]. Geographically, the research was conducted primarily in Northern Europe and the USA. The target populations mainly included older adults and people living with COPD [48,50,53], CHF [48,50], CVD [45], type 2 diabetes [47,51,54,55], and complex multimorbidity [46,49,56,57,59,60] or frailty [52,58].

**Figure 2:**
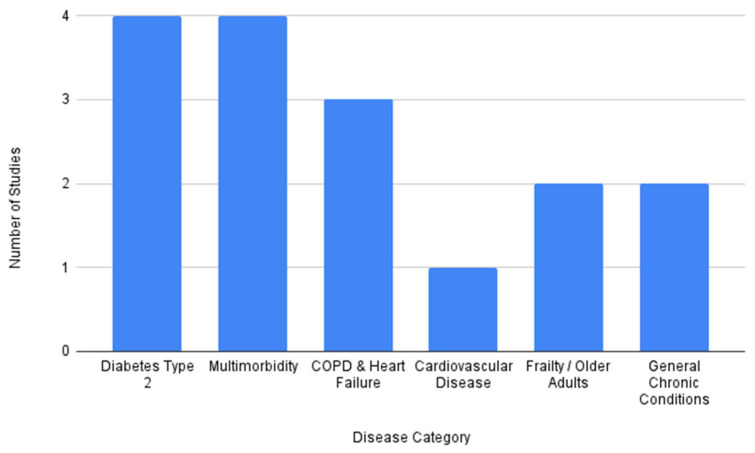
Distribution of included studies by disease category (*n* = 16).

The distribution of included studies by primary chronic condition is visualized in Figure 2, highlighting a strong focus on type 2 diabetes and multimorbidity.

Across studies, digital interventions were predominantly multicomponent, extending beyond basic telemonitoring toward integrated digital ecosystems. These included interactive platforms supporting two-way messaging and shared digital health plans [49,51], patient portals and personal health records (PHRs) enabling information access and exchange [46], and cloud-based or virtual clinic models integrating devices, messaging, video, and data repositories [57]. A consistent pattern was the integration of digital tools with structured human support, such as nurse-led telephone or video coaching [49,56], asynchronous support centers linked to connected devices [61], and telehealth-delivered collaborative goal-setting interventions [58]. Emerging work also highlights design-oriented innovations, including AI-enabled tools to support values elicitation and preparation for person-centered clinical encounters [53] (Table 1).

### 3.2. Operationalization of Person-Centered Principles in Digital Environments

The studies demonstrated that digital tools operationalize PCC through several key mechanisms. The elicitation of patient narratives and values was facilitated via digital worksheets and messaging apps, allowing patients to share “what matters to them” beyond clinical metrics [51,52,57]. Co-creation of health plans was a recurring theme, where digital platforms served as a shared space for documenting goals and negotiating care strategies [57,58]. Furthermore, the safeguarding of the partnership was achieved through continuous access to data and professionals, shifting the patient’s role to an active partner [50,54]. While these tools aim for individualization, some evidence suggests a persistent gap in addressing sex and gender-specific design within these digital trials [61].

### 3.3. Clinical, Psychosocial, and Systematic Outcomes

The impact of these interventions was generally favorable, although effects varied by condition, outcome type, and study design. Evidence from systematic reviews and smaller trials suggests improvements in clinical indicators, most consistently glycemic control (HbA1c) in diabetes-focused interventions, alongside signals for improved self-management and related risk factors [45,47,55]. In contrast, findings from larger pragmatic evaluations were more mixed: for example, digital PCC combined with structured support did not significantly improve the primary composite endpoint in intention-to-treat analyses, even though short-term gains in self-efficacy were observed in per-protocol analyses [48]. At the service level, proactive person-centered models evaluated using routine data were associated with reduced emergency admissions and inpatient bed days in frail multimorbid populations [58].

From a psychosocial perspective, digital PCC approaches consistently emphasized empowerment, autonomy, and person-relevant goal pursuit, supported by partnership-based practices such as shared decision-making and collaborative goal setting [52,54,57]. Qualitative accounts further described digital PCC as offering “side door” access to care, enabling timely contact and continuity while reducing the practical burden of in-person visits [50]. Regarding healthcare utilization and costs, remote monitoring services showed higher engagement among chronic patients and trends toward lower healthcare charges, although these findings were not always based on controlled designs [60]. Across studies, feasibility and acceptability were frequently reported as high in pilots and qualitative work [54,55], but sustained impact appears dependent on addressing persistent digital access and use disparities linked to multimorbidity burden, age, literacy, and socio-economic factors [47,59] (Table 2).

### 3.4. Critical Evaluation of Articles

Table 3 presents the critical appraisal of the included systematic reviews [45,46,47] using the JBI Critical Appraisal Checklist for Systematic Reviews and Research Syntheses, to support methodological development, conduct, and reporting within an umbrella review approach. This checklist evaluates key domains of review quality, including the clarity of the review question, appropriateness of inclusion criteria, rigor and comprehensiveness of the search strategy, suitability of critical appraisal and synthesis methods, and strategies to minimize bias. Overall, the included reviews performed strongly across core items (Q1–Q4, Q7 and Q8), indicating generally robust review design and conduct. However, some variability was identified in appraisal-related processes: Mitchell et al. [46] did not meet items related to appraisal criteria and independent appraisal by multiple reviewers (Q5, Q6), while Brands et al. [45] provided unclear information for independent critical appraisal (Q6). Notably, none of the reviews adequately assessed publication bias (Q9), representing a shared methodological limitation. Collectively, this appraisal provides a transparent basis for judging the trustworthiness of the included systematic reviews and supports their interpretation within the current umbrella review.

Table 4 presents the critical appraisal of the included randomized controlled trials [48,49,55,58] using the revised JBI Critical Appraisal Tool for the Assessment of Risk of Bias for Randomized Controlled Trials (RCTs) [38]. This tool uses signaling questions to evaluate risk of bias across key domains of validity, including internal validity (selection/allocation, performance, detection/measurement, and attrition bias) and statistical conclusion validity (e.g., appropriateness of analyses and adherence to intention-to-treat principles). Overall, the trials demonstrated strengths in statistical analysis (Q12), and most accounted for trial design considerations (Q13), although Berntsen et al. [58] did not meet several design and allocation safeguards (Q1, Q2 and Q13). Across studies, important limitations were identified in blinding: participant blinding (Q4) and blinding of those delivering the intervention (Q5) were consistently not achieved, and outcome assessor blinding (Q7) was frequently unclear. Selection and allocation procedures were variably reported, with allocation of concealment often unclear (Q2) and baseline comparability unclear in Kearney et al. [49] (Q3). Reporting and handling of follow-up also varied (Q10), with Carter et al. [55] not adequately addressing incomplete follow-up. Collectively, these findings provide a transparent assessment of methodological rigor and support interpretation of the RCT evidence with appropriate consideration of identified risks of bias.

Table 5 presents the critical appraisal of the included qualitative studies [50,51,52,53,54] using the JBI Critical Appraisal Checklist for Qualitative Research. This appraisal is aligned with the JBI methodological guidance for qualitative evidence synthesis using meta-aggregation, as described by Lockwood et al. [64], which is grounded in pragmatism and emphasizes the transparent aggregation of qualitative findings to inform practice and policy. The checklist evaluates methodological rigor through assessment of congruity between the philosophical perspective, research methodology, data collection, analysis, and interpretation, as well as researcher reflexivity, ethical considerations, and representation of participants’ voices. Overall, the included studies demonstrated strong methodological coherence, with consistent “Yes” ratings across key domains related to research design, data collection, analysis, and alignment of conclusions with findings (Q2–Q5, Q8–Q10). However, several studies provided limited or unclear reporting on researcher positioning and reflexivity (Q6, Q7), indicating a common methodological limitation. Barenfeld et al. [50] most comprehensively addressed these reflexivity criteria. Despite these limitations, all studies were judged to be of sufficient methodological quality to support credible meta-aggregation and meaningful contribution to the qualitative synthesis.

Table 6 presents the critical appraisal of the included analytical cross-sectional study by Barker et al. [40] using JBI Critical Appraisal Checklist for Analytical Cross-Sectional Studies. This tool evaluates methodological quality across key domains, including clarity of inclusion criteria, detailed description of the study population and setting, validity and reliability of exposure and outcome measurements, identification and management of confounding factors, and appropriateness of statistical analysis. Overall, the study demonstrated good methodological quality, with clear inclusion criteria, well-described study subjects and setting, valid measurement of exposure and outcomes, identification of potential confounders, and appropriate strategies to address confounding (Q1, Q2, Q4–Q6, and Q8). However, some aspects were insufficiently reported, particularly regarding the validity and reliability of exposure measurement and outcome assessment (Q3 and Q7), which were rated as unclear. Despite these limitations, the study was judged to be of adequate methodological rigor to support its inclusion and contribution to the evidence synthesis.

Table 7 presents the critical appraisal of the included cohort study by Petersen et al. [60] using the Critical Appraisal Skills Programme (CASP) Cohort Study Checklist. This tool assesses methodological quality across key domains, including whether the study addressed a clearly focused issue, the acceptability of cohort recruitment, the validity of exposure and outcome measurement, identification of confounding factors, adequacy of follow-up, clarity and precision of results, and the applicability of findings to practice.

Overall, the study showed mixed methodological quality. Several core domains were rated positively, including a clearly focused research question and adequate measurement of exposure and outcomes (Q1, Q3–Q5), as well as reporting sufficient information on result precision (Q8). However, weaknesses were identified in cohort recruitment and in the reporting of study results (Q2 and Q7), which were rated “No.” In addition, multiple domains were judged “Unclear” due to insufficient reporting (Q6, Q9–Q12), particularly regarding follow-up adequacy, confidence in the results, and the broader applicability and implications for practice. Despite these limitations, the study was considered informative, but its findings should be interpreted with caution because several critical aspects were not reported transparently.

Table 8 presents the critical appraisal of the included mixed methods studies by Kang et al. [57] and de Jong et al. [56] using the Mixed Methods Appraisal Tool (MMAT). The tool evaluates the clarity of research questions, the appropriateness of the mixed methods design, the quality of the qualitative and quantitative components, the integration of study components, and the adequacy of interpretation of the combined results. Overall, both studies demonstrated generally good methodological quality, with clear research questions and appropriate use of mixed methods approaches (S1, S2, 1.1–1.4, 4.1, 4.3, 4.5, 5.1–5.3). However, both studies showed limitations in certain quantitative domains (4.2), which were rated “No.” Some aspects were insufficiently reported, particularly regarding elements of integration and interpretation (5.4 and 5.5), and in one case, specific methodological details (1.5 and 4.4), which were rated as “Unclear.” Despite these limitations, both studies were considered methodologically sound overall and suitable for inclusion, although some findings should be interpreted with caution due to incomplete reporting in selected domains.

## 4. Discussion

Our synthesis shows that digital health interventions meaningfully reshape the operationalization of PCC in chronic disease management. Across the included studies, digital tools frequently function as a bridge to core principles such as patient narratives, partnership, and shared documentation, facilitating a shift from episodic clinic encounters toward more continuous and collaborative health management [48,50,56,60,65,66]. This was particularly evident in interventions that combined two-way messaging, visualization of patient self-ratings, and shared access to documentation, such as research by authors Ali et al. [48], and in models of virtual outpatient care and telemonitoring where data from monitoring devices flow into a shared record and trigger timely team support [48,56,67]. In this sense, platforms in our sample did not operate as passive repositories but rather as enabling infrastructures that support “side-door” access to care, greater responsiveness, and continuity of the therapeutic relationship. Qualitative findings further reinforce this interpretation, as patients described ePCC as being “welcomed through the side door”, which strengthened feelings of safety, inclusion in the health system, and the capacity for self-management, especially when traditional routes to care were constrained [50]. To enhance conceptual clarity, our synthesis identifies three core digital PCC mechanisms: (1) patient narratives, enabled by AI simulators and messaging to elicit ‘what matters’; (2) shared decision-making, supported by real-time data visualization that allows patients and providers to jointly adjust goals; and (3) continuous partnership, facilitated by asynchronous support that bridges the gap between clinic visits.

When mapping our findings to PCC mechanisms, three recurring processes explain why digital interventions often improve care experiences and processes. First, they enhance situational awareness through self-ratings, monitoring devices, and trend visualizations, helping patients make sense of their condition and detect deterioration earlier [50,57,66]. Second, they strengthen partnership via two-way communication, asynchronous support, and tailored coaching, enabling relationship continuity beyond face-to-face visits [48,51,55]. Third, they promote shared documentation through co-created plans, personal health records, and structured summaries of goals and values, which enable “shared tracking” of agreements and greater transparency [45,48,52,68].

At the same time, our synthesis indicates that a digital component alone is often insufficient for sustained outcomes. In the randomized trial, improved general self-efficacy was observed only short-term in a per-protocol analysis, and the effect was not maintained at six months; there were also no differences in hospitalizations or the composite endpoint in the intention-to-treat analysis [48]. A similar signal appears in telephone-delivered PCC without a digital portal, where the primary composite endpoint did not differ, although there was a reduced risk of clinically important deterioration in self-efficacy [69]. Taken together, these findings support the interpretation that digital PCC more readily improves process outcomes and care experiences, whereas long-term clinical effects require more than technology, particularly sustained relational support and clear organizational pathways for integrating patient-generated data into clinical decision-making [53,54,70]. This is consistent with models where digital infrastructures were tightly coupled with nurses, coaches, or navigation teams. For example, a telehealth portal with regular nurse-led videoconferences was associated with improvements in metabolic indicators and self-management [55], and an mHealth coaching program demonstrated communication strategies that systematically support autonomy, reflection, and goal adaptation [51]. Similarly, programs combining 24/7 support and alert analytics suggest that people with chronic conditions engage more intensively, with the workload implications for clinical staff and the need for sustainable staffing and reimbursement models [60].

The success of digital PCC also depends on reducing technological and social gaps. Nationally representative data indicate that health tracking and information sharing with clinicians vary by sociodemographic characteristics and access to the internet, devices, and online medical records, which can limit the reach of collaborative care [59]. Conceptual contributions further warn that without participatory, diversity-sensitive design, eHealth solutions may exclude vulnerable groups and inadvertently widen inequities [71]. In this context, it is also notable that app-based RCTs for chronic conditions often inadequately report sex and gender analyses, limiting the assessment of equity and differential effects [61]. Reviews focusing on patient portals and personal health records report the most consistent benefits for engagement, knowledge, adherence, self-management, and some laboratory parameters, while effects on quality of life are less consistent, aligning with our observation of heterogeneity in interventions and outcomes [45].

Based on this synthesis, we recommend that future research and implementation efforts more clearly define and measure the “active ingredients” of PCC in digital contexts. This includes consistent assessment of whether patient narratives are elicited, whether a co-created and patient-accessible plan is present, how shared data use and ownership are governed, and whether responsive team processes translate patient-generated data into meaningful actions. Alongside clinical endpoints, implementation outcomes such as acceptability, appropriateness, costs, and sustainability should be routinely measured, with transparent reporting of differences across social and demographic groups to strengthen real-world transferability [47,59]. A key research priority is to test hybrid models that explicitly combine digital tools with sustained “high-touch” relational support, as our sample suggests that technology without structured support is less likely to produce stable behavioral and clinical changes [48,51,55]. Although our review focused on chronic disease management, the identified PCC mechanisms, such as eliciting patient narratives and ‘what matters’, are highly applicable to palliative and terminal care settings, where maintaining personhood and dignity is paramount.

The limitations of this article reflect both the nature of the existing literature and the scope of our synthesis. The included evidence base is methodologically heterogeneous, encompassing randomized trials, observational studies, qualitative research, systematic and scoping reviews, and conceptual contributions, which complicates direct comparisons and prevents robust attribution of effects to specific technological features. Interventions vary substantially in intensity, duration, level of integration with health information systems, and the presence of human support, making it difficult to isolate mechanisms of impact. In addition, the lack of long-term longitudinal data and inconsistent outcome reporting, particularly for implementation and equity dimensions, limits conclusions about sustainability and effects on disparities. Many studies implicitly select for higher digital access and literacy, potentially underestimating barriers in more vulnerable populations [59,71]. An additional limitation concerns the conceptual variability of PCC across included studies. Although all eligible articles explicitly referred to person-centered, patient-centered, individualized, or personalized care, the underlying definitions, theoretical frameworks, and operationalizations of PCC varied considerably. As a result, what constituted “PCC” design or implementation differed across interventions, limiting comparability and synthesis of PCC components. Moreover, this review focused on identifying and describing digital interventions that explicitly incorporated PCC, rather than evaluating the depth, quality, or fidelity of PCC implementation. The analysis therefore relied on how PCC was reported and framed by study authors, which may not fully capture how PCC principles were enacted in practice. Relatedly, the decision to include only studies in which PCC terminology was explicitly stated may have resulted in the exclusion of digital interventions that incorporate PCC principles implicitly but do not label them as such. While this approach ensured conceptual clarity and alignment with the research question, it may have narrowed the scope of identified interventions. Finally, because part of the evidence includes reviews and conceptual papers, these sources are valuable for interpreting mechanisms and trends but cannot substitute for empirical evaluations of effectiveness in comparable settings. While our final sample includes 16 records, this relatively small number reflects the current state of the field, where many digital interventions may apply person-centered principles implicitly but fail to use explicit PCC terminology. Our strict eligibility criteria were necessary to ensure conceptual clarity and to specifically evaluate how PCC is formally recognized and operationalized in digital health. Furthermore, these studies represent a wide range of methodologies, from RCTs to qualitative syntheses, providing a high-quality, albeit focused, evidence base.

In conclusion, our synthesis supports the view that digitalization can strengthen PCC when digital tools genuinely enable narratives, partnership, and shared documentation and are embedded within organizational models that support the use of patient-generated data and provide relational continuity. It is essential that digital PCC does not lead to depersonalization but instead extends the therapeutic relationship and access to care. We emphasize that successful implementation will depend mostly on systematic education and training for nurses and other team members on integrating PCC principles into digital encounters, developing digital communication skills, interpreting patient-generated data, and safely co-managing shared documentation. Without these competencies and organizational support for digital partnership work, there is a risk that digital tools will remain technological add-ons rather than catalysts for deeper, more accessible patient–provider partnerships.

## 5. Conclusions

In conclusion, this review demonstrates that digital health interventions can meaningfully strengthen PCC in chronic disease management when they function as active enablers of patient narratives, sustained partnership, and shared documentation, rather than mere information repositories. Digital tools are most effective when they prioritize patient values over clinical metrics, leading to consistent benefits in care processes and patient experiences, such as improved access to support and greater situational awareness. However, effects on long-term clinical outcomes remain inconsistent. The persistent ‘digital divide’ and the need for durable clinical change suggest that technology alone is insufficient; it must be coupled with organizational shifts, structured “high-touch” clinical support, and professional training.

Future work should move beyond evaluating technologies as standalone tools and instead test hybrid care models. Research must adopt clearer operational definitions of PCC in digital contexts and consistently measure implementation outcomes, equity dimensions, and longer follow-up periods. Ultimately, future implementation must move away from ‘technology-first’ approaches toward ‘person-first’ digital health ecosystems. From a practice perspective, successful scale-up will depend on robust organizational readiness, interoperable information systems, and systematic education of healthcare professionals in applying PCC principles during digital encounters.

## Figures and Tables

**Table 1:** Synthesis of results from the literature: digital interventions and person-centered care (PCC).

Author, Year	Aim	Methodology	Population	Digital Intervention	PCC (Person-Centered Care/Person-Centered Principles)	Outcomes
Ali et al. [48], Sweden	To evaluate the effects of PCC delivered via a digital platform and telephone support on self-efficacy and hospitalization.	Multicenter RCT (PROTECT) comparing intervention vs. standard care.	222 adults with COPD or CHF; mean age 70.8 years.	Combined digital platform and structured telephone support; messaging, shared health plans, and symptom self-ratings.	Patient narratives, co-creating health plans, shared decision-making, and involving informal carers.	No significant differences in composite endpoint. Per-protocol analysis showed improved self-efficacy in 3 months.
Barenfeld et al. [50], Sweden	To explore experiences of a PCC e-health intervention in patients with COPD or CHF.	Qualitative research article (Grounded Theory).	12 purposively sampled patients from the PROTECT project; mean age 71.4.	Interactive platform (messaging, trend graphs, personal notes) and structured PCC telephone support.	Patient narratives, partnership/shared decision-making, and shared documentation (health plan).	Core category: “Being welcomed through the side door,” describing remote access and strengthened patient position.
Berntsen et al. [58], Norway	To evaluate the proactive care (PACT) model combining PCC, integrated, and proactive care for frail elderly.	Pragmatic prospective propensity score–matched controlled trial using EHR data.	439 PACT patients and 779 matched controls; frail multimorbid adults >60.	Cross-organizational team model; intervention evaluated using routine hospital EHR data.	“What matters to you?” goal-oriented care, co-created goals, and proactive risk management.	Reduced emergency admissions and inpatient bed days in the PACT group.
Brands et al. [45]	To systematically review the effects of PCC digital health records (PHRs) on outcomes for chronic conditions.	Systematic review of 81 studies.	1,639,556 participants across chronic conditions (primarily diabetes and CVD).	Patient portals, PHRs, and mHealth apps enabling two-way health information exchange.	Access to data, two-way communication, active engagement, and self-management support.	Beneficial effects on healthcare utilization, patient-reported outcomes, and clinical parameters (HbA1c).
Carter et al. [55], USA	To describe the design and outcomes of a pilot self-management intervention for African Americans with type 2 diabetes.	Pilot randomized controlled intervention study.	47 urban African American adults with type 2 diabetes.	Telehealth portal with peripherals (scale, BP cuff, glucometer) and biweekly nurse video conferences.	Culturally competent, individualized action plans based on patient needs/preferences.	Significant improvement in HbA1c (9.0 to 6.82) and BMI in the treatment group.
de Jong et al. [56], The Netherlands	To assess the feasibility of a “Virtual Outpatient Clinic” (VOC) for integrating self-monitoring and communication.	Proof-of-concept feasibility study.	20 adult patients with broad disease spectrum and comorbidities.	Cloud-based service with 6 tools: measuring devices, video apps, messaging, and PHR integration.	Data ownership, self-monitoring, tailored tool use, and empowerment through health awareness.	High feasibility and positive user experience; increased awareness of health status and control.
Griffin & Chung [59], USA	To examine the state of health tracking and information sharing among patients with chronic conditions.	Cross-sectional secondary data analysis (HINTS survey).	2439 adults with one or more chronic conditions.	Smartphones, tablets, electronic monitoring devices, and online medical records.	Improving patient-clinician communication and enabling patients to track and share “lived” health data.	Patients with 3+ conditions had higher odds of digital sharing (OR 3.61). Tech access gaps persist.
Kang et al. [57], USA	To determine the feasibility of a PCC goal setting system (MyGoals) via telehealth and hybrid formats.	Mixed-method feasibility study.	17 adults with one or more chronic conditions.	MyGoals intervention delivered via Zoom or hybrid format using structured worksheets.	Collaborative goal setting based on narratives and personally meaningful goals.	High credibility, satisfaction, and person-centeredness of goals; high achievement of change objectives.
Kearney et al. [49], USA	To compare effectiveness of high-touch, high-tech, and usual care for adults with multiple chronic conditions and assess implementation.	Pragmatic randomized controlled trial with implementation strategies.	Adults ≥21 years with ≥1 chronic physical condition plus ≥1 additional physical or behavioral condition; Medicaid or dual-eligible; recently discharged.	High-tech digital care platform (iPhone) with video visits, condition-specific text check-ins, alerts, and digital health tools.	Stakeholder-engaged, patient-centered outcomes (PCORI); patient partners shaping procedures; focus on activation, tailoring, low burden, shared decision-making.	Primary: 30- and 90-day readmissions, health status, patient activation. Secondary: quality of life, satisfaction, care use; qualitative implementation findings.
Kim et al. [51], USA	To identify patterns of PCC used by coaches in an mHealth diabetes prevention program.	Qualitative study.	30 Noom Coach DPP users (aged 65–74).	Smartphone-based logging/tracking and continuous one-to-one coaching via in-app chat.	Fostering autonomy, jointly setting measurable goals, and tailoring guidance to user lifestyle.	Identified four PCC strategies; mHealth features supported situational awareness and user autonomy.
Mitchell et al. [46]	To synthesize how PCC methods are used in designing ICTs for chronic disease management and healthy behaviors.	Systematic review (PRISMA); descriptive thematic synthesis.	57 studies involving patients as end-users across multiple conditions (ages 5–78).	Health ICTs including mobile apps, web platforms, software, personal assistants, and serious games for self-management and support.	Person-centered design via end-user involvement (focus groups, interviews, workshops, surveys); emphasis on tailoring, usability, accessibility, and communication.	Thematic outcomes: participant experience, technological requirements, behavioral/knowledge, and social components; limited theory-driven design.
Mokaya et al. [47]	To examine clinical and PCC implementation outcomes of mHealth for type 2 diabetes in LMICs.	Systematic review (30 studies).	Adults with type 2 diabetes in low- and middle-income countries.	SMS text messages (most common), mobile apps, and telemedicine approaches.	Focus on feasibility, acceptability, and appropriateness of interventions within user context.	Improved HbA1c and fasting blood glucose; highlighted barriers like literacy and connectivity.
Petersen et al. [62], USA	To examine staff utilization and costs of a person-centered remote monitoring model (ImagineCare).	Cohort study	2894 employees (376 with chronic conditions).	Bluetooth devices paired with a mobile app and 24/7 care support center.	Documentation of patient goals/preferences; tailored device allocation and 24/7 asynchronous communication.	Chronic patients showed higher engagement. Trends toward 16–20% reduction in healthcare charges.
Rooper et al. [52], USA	To identify design considerations for technology that facilitate values elicitation in MCC patients.	Original qualitative user-centered design study.	18 workshop participants (clinicians and adults with MCC).	TES prototypes: mobile worksheets and an AI chatbot “PCP simulator.”	Eliciting “what matters,” translating values into care priorities, and supporting appointment preparation.	Design guidance produced: focus on actionable plans, low-burden activities, and EHR integration.
Smaradottir & Fensli [53], Norway/Denmark	To evaluate benefits and constraints of telemedicine in patient-centered care teams and identify barriers to collaborative work.	Qualitative study (interviews and observations, 2017–2019).	27 informants (health professionals, technical staff, and patient representatives) involved in PCC team models for chronic conditions (mainly COPD).	Telemedicine systems for remote follow-up (home measurements, questionnaires, digital communication), run alongside EHRs.	Patient-centered teamwork model; focus on collaboration, information sharing, and support for person-centered telemedicine follow-up.	Telemedicine systems were usable but poorly integrated; fragmented information flow and limited interoperability hindered PCC teamwork; lack of patient access to stored information identified as a key limitation.
Wildevuur et al. [54], The Netherlands	To construct a theoretical framework for ICT-enabled partnership in diabetes management.	Qualitative research article: inductive case study.	Patients with type 1 diabetes and healthcare professionals.	Wearable artificial pancreas system with data transmission to a shared portal.	Patient as equal partner, shared data analysis, reciprocity, and trust in technology.	Themes: self-managing, shared data analysis, and experiencing partnership; improved carefree living.

**Table 2:** Summary of key findings and supporting evidence.

Outcome Category	Key Observations	Supporting Articles
Clinical Efficacy	Improved metabolic control, most consistently HbA1c (and related risk factors in some studies).	[45,47,55]
Psychosocial Impact	Improved self-efficacy, empowerment/autonomy, and perceived security/continuity.	[49,51,52,55,57]
Care Process	PCC operationalized via co-created goals/health plans, shared documentation, and partnership-based communication.	[49,51,53,55,56,58]
Health Utilization	Signals of reduced acute care use/costs in some models (mixed evidence across designs).	[46,49,59,61]
Implementation	High feasibility/acceptability in pilots; outcomes depend on digital literacy/access and system integration/interoperability.	[48,54,57,58,60]

**Table 3:** Critical assessment of systematic reviews (SR) [63].

Checklist Question	Mokaya et al. [47]	Mitchell et al. [46]	Brands et al. [45]
Q1 ^1^	Yes	Yes	Yes
Q2 ^2^	Yes	Yes	Yes
Q3 ^3^	Yes	Yes	Yes
Q4 ^4^	Yes	Yes	Yes
Q5 ^5^	Yes	No	Yes
Q6 ^6^	Yes	No	Unclear
Q7 ^7^	Yes	Yes	Yes
Q8 ^8^	Yes	Yes	Yes
Q9 ^9^	No	No	No
Q10 ^10^	Unclear	Yes	Yes
Q11 ^11^	Yes	Yes	Yes

^1^ Is the review question clearly and explicitly stated? ^2^ Were the inclusion criteria appropriate for the review question? ^3^ Was the search strategy appropriate? ^4^ Were the sources and resources used to search for studies adequate? ^5^ Were the criteria for appraising studies appropriate? ^6^ Was critical appraisal conducted by two or more reviewers independently? ^7^ Were there methods to minimize errors in data extraction? ^8^ Were the methods used to combine studies appropriate? ^9^ Was the likelihood of publication bias assessed? ^10^ Were recommendations for policy and/or practice supported by the reported data? ^11^ Were the specific directives for new research appropriate?

**Table 4:** Critical assessment of RCT [38].

Checklist Question	Kearney et al. [49]	Carter et al. [55]	Ali et al. [48]	Berntsen et al. [58]
Q1 ^1^	Yes	Yes	Yes	No
Q2 ^2^	Unclear	Unclear	Yes	No
Q3 ^3^	Unclear	Yes	Yes	Yes
Q4 ^4^	No	No	No	No
Q5 ^5^	No	No	No	No
Q6 ^6^	Unclear	Yes	Yes	Yes
Q7 ^7^	Unclear	Unclear	Unclear	Yes
Q8 ^8^	Yes	No	Yes	Yes
Q9 ^9^	Unclear	Unclear	Yes	Yes
Q10 ^10^	Unclear	No	Yes	Yes
Q11 ^11^	Yes	No	Yes	N/A
Q12 ^12^	Yes	Yes	Yes	Yes
Q13 ^13^	Yes	Yes	Yes	No

^1^ Was true randomization used for assignment of participants to treatment groups? ^2^ Was allocation to treatment groups concealed? ^3^ Were treatment groups similar at the baseline? ^4^ Were participants blind to treatment assignment? ^5^ Were those delivering the treatment blind to treatment assignment? ^6^ Were treatment groups treated identically other than the intervention of interest? ^7^ Were outcome assessors blind to treatment assignment? ^8^ Were outcomes measured in the same way for treatment groups? ^9^ Were outcomes measured in a reliable way ^10^ Was follow up complete and if not, were differences between groups in terms of their follow up adequately described and analysed? ^11^ Were participants analysed in the groups to which they were randomized? ^12^ Was appropriate statistical analysis used? ^13^ Was the trial design appropriate and any deviations from the standard RCT design (individual randomization, parallel groups) accounted for in the conduct and analysis of the trial?

**Table 5:** Critical assessment of qualitative study [64].

Checklist Question	Wildevuur et al. [54]	Rooper et al. [52]	Kim et al. [51]	Barenfeld et al. [50]	Smaradottir & Fensli [53]
Q1 ^1^	Yes	Yes	Unclear	Yes	Unclear
Q2 ^2^	Yes	Yes	Yes	Yes	Yes
Q3 ^3^	Yes	Yes	Yes	Yes	Yes
Q4 ^4^	Yes	Yes	Yes	Yes	Yes
Q5 ^5^	Yes	Yes	Yes	Yes	Yes
Q6 ^6^	Unclear	Unclear	Unclear	Yes	Unclear
Q7 ^7^	Unclear	Unclear	Unclear	Yes	Unclear
Q8 ^8^	Yes	Yes	Yes	Yes	Yes
Q9 ^9^	Yes	Yes	Yes	Yes	Yes
Q10 ^10^	Yes	Yes	Yes	Yes	Yes

^1^ Is there congruity between the stated philosophical perspective and the research methodology? ^2^ Is there congruity between the research methodology and the research question or objectives? ^3^ Is there congruity between the research methodology and the methods used to collect data? ^4^ Is there congruity between the research methodology and the representation and analysis of data? ^5^ Is there congruity between the research methodology and the interpretation of results? ^6^ Is there a statement locating the researcher culturally or theoretically? ^7^ Is the influence of the researcher on the research, and vice- versa, addressed? ^8^ Are participants, and their voices, adequately represented? ^9^ Is the research ethical according to current criteria or, for recent studies, and is there evidence of ethical approval by an appropriate body? ^10^ Do the conclusions drawn in the research report flow from the analysis, or interpretation, of the data?

**Table 6:** Critical assessment of cross-sectional study [40].

Checklist Question	Griffin & Chung [59]
Q1 ^1^	Yes
Q2 ^2^	Yes
Q3 ^3^	Unclear
Q4 ^4^	Yes
Q5 ^5^	Yes
Q6 ^6^	Yes
Q7 ^7^	Unclear
Q8 ^8^	Yes

^1^ Were the criteria for inclusion in the sample clearly defined? ^2^ Were objective, standard criteria used for measurement of the condition? ^3^ Was the exposure measured in a valid and reliable way? ^4^ Were the outcomes measured in a valid and reliable way? ^5^ Were confounding factors identified? ^6^ Were strategies to deal with confounding factors stated? ^7^ Was appropriate statistical analysis used? ^8^ Were the study subjects and the setting described in detail?

**Table 7:** Critical assessment of cohort study.

Checklist Question	Petersen et al. [60]
Q1 ^1^	Yes
Q2 ^2^	No
Q3 ^3^	Yes
Q4 ^4^	Yes
Q5 ^5^	Yes
Q6 ^6^	Unclear
Q7 ^7^	No
Q8 ^8^	Yes
Q9 ^9^	Unclear
Q10 ^10^	Unclear
Q11 ^11^	Unclear
Q12 ^12^	Unclear

^1^ Did the study address a clearly focused issue? ^2^ Was the cohort recruited in an acceptable way? ^3^ Was the exposure accurately measured to minimise bias? ^4^ Was the outcome accurately measured to minimise bias? ^5^ (a) Have the authors identified all important confounding factors? (b) Have they taken account of the confounding factors in the design and/or analysis? ^6^ (a) Was the follow up of subjects complete enough? (b) Was the follow up of subjects long enough? ^7^ What are the results of this study? ^8^ How precise are the results? ^9^ Do you believe the results? ^10^ Can the results be applied to the local population? ^11^ Do the results of this study fit with other available evidence? ^12^ What are the implications of this study for practice?

**Table 8:** Critical assessment of mixed method study.

Checklist Question	Kang et al. [57]	de Jong et al. [56]
S1 ^1^	Yes	Yes
S2 ^2^	Yes	Yes
1.1 ^3^	Yes	Yes
1.2 ^4^	Yes	Yes
1.3 ^5^	Yes	Yes
1.4 ^6^	Yes	Yes
1.5 ^7^	Yes	Unclear
2.1 ^8^	Yes	Yes
2.2 ^9^	Yes	Yes
2.3 ^10^	Yes	Yes
2.4 ^11^	Yes	Yes
2.5 ^12^	Yes	Yes
3.1 ^13^	Yes	Yes
3.2 ^14^	Yes	Yes
3.3 ^15^	Yes	Yes
3.4 ^16^	Yes	Yes
3.5 ^17^	Yes	Yes
4.1 ^18^	Yes	Yes
4.2 ^19^	No	No
4.3 ^20^	Yes	Yes
4.4 ^21^	Unclear	Yes
4.5 ^22^	Yes	Yes
5.1 ^23^	Yes	Yes
5.2 ^24^	Yes	Yes
5.3 ^25^	Yes	Yes
5.4 ^26^	Unclear	Unclear
5.5 ^27^	Unclear	Unclear

^1^ Are there clear research questions? ^2^ Do the collected data allow to address the research questions? ^3^ Is the qualitative approach appropriate to answer the research question? ^4^ Are the qualitative data collection methods adequate to address the research question? ^5^ Are the findings adequately derived from the data? ^6^ Is the interpretation of results sufficiently substantiated by data? ^7^ Is there coherence between qualitative data sources, collection, analysis and interpretation? ^8^ Is randomization appropriately performed? ^9^ Are the groups comparable at baseline? ^10^ Are there complete outcome data? ^11^ Are outcome assessors blinded to the intervention provided? ^12^ Did the participants adhere to the assigned intervention? ^13^ Are the participants representative of the target population? ^14^ Are measurements appropriate regarding both the outcome and intervention (or exposure)? ^15^ Are there complete outcome data? ^16^ Are the confounders accounted for in the design and analysis? ^17^ During the study period, is the intervention administered (or exposure occurred) as intended? ^18^ Is the sampling strategy relevant to address the research question? ^19^ Is the sample representative of the target population? ^20^ Are the measurements appropriate? ^21^ Is the risk of nonresponse bias low? ^22^ Is the statistical analysis appropriate to answer the research question? ^23^ Is there an adequate rationale for using a mixed methods design to address the research question? ^24^ Are the different components of the study effectively integrated to answer the research question? ^25^ Are the outputs of the integration of qualitative and quantitative components adequately interpreted? ^26^ Are divergences and inconsistencies between quantitative and qualitative results adequately addressed? ^27^ Do the different components of the study adhere to the quality criteria of each tradition of the methods involved?

## Data Availability

No new data were created or analyzed in this study. Data sharing is not applicable to this article as all analyzed sources and findings are included within the manuscript.

## Supplementary Materials

The following supporting information can be downloaded at: https://www.mdpi.com/article/10.3390/healthcare14081048/s1, Table S1: Preferred Reporting Items for Systematic reviews and Meta-Analyses extension for Scoping Reviews (PRISMA-ScR) Checklist.

## Abbreviations

The following abbreviations are used in this manuscript:

PCC	Person-Centered Care
PRISMA	Preferred Reporting Items for Systematic Reviews and Meta-Analyses
PIO	Population, Intervention, Outcomes
JBI	Joanna Briggs Institute
RCT	Randomized Controlled Trial
PROTECT	Person-Centred Care for Chronic Obstructive Pulmonary Disease and Chronic Heart Failure
COPD	Chronic Obstructive Pulmonary Disease
CHF	Chronic Heart Failure
CVD	Cardiovascular Disease
PHR	Personal Health Record
ICT	Information and Communication Technology
mHealth	Mobile Health
eHealth	Electronic Health
AI	Artificial Intelligence
GenAI	Generative Artificial Intelligence
EHR	Electronic Health Record
HINTS	Health Information National Trends Survey
LMICs	Low- and Middle-Income Countries
HbA1c	Glycated Hemoglobin
BMI	Body Mass Index
DPP	Diabetes Prevention Program
TES	Technology-Enabled System
APC	Article Processing Charge
WHO	World Health Organization
PCORI	Patient-Centered Outcomes Research Institute
VOC	Virtual Outpatient Clinic
